# Measuring Sharp Waves and Oscillatory Population Activity With the Genetically Encoded Calcium Indicator GCaMP6f

**DOI:** 10.3389/fncel.2019.00274

**Published:** 2019-06-19

**Authors:** Pinggan Li, Xinling Geng, Huiyi Jiang, Adam Caccavano, Stefano Vicini, Jian-young Wu

**Affiliations:** ^1^Department of Pediatric Neurology, Sun Yat-sen Memorial Hospital, Sun Yat-sen University, Guangzhou, China; ^2^Department of Neuroscience, Georgetown University, Washington, DC, United States; ^3^School of Biomedical Engineering, Capital Medical University, Beijing, China; ^4^Department of Pediatrics, The First Hospital of Jilin University, Changchun, China; ^5^Department of Pharmacology and Physiology, Georgetown University, Washington, DC, United States

**Keywords:** GCaMP, calcium signals, hippocampal slice, voltage-sensitive dye imaging, local field potential recordings, theta oscillation, hippocampal sharp wave

## Abstract

GCaMP6f is among the most widely used genetically encoded calcium indicators for monitoring neuronal activity. Applications are at both the cellular and population levels. Here, we explore two important and under-explored issues. First, we have tested if GCaMP6f is sensitive enough for the detection of population activity with sparse firing, similar to the sensitivity of the local field potential (LFP). Second, we have tested if GCaMP6f is fast enough for the detection of fast network oscillations critical for the encoding and consolidation of memory. We have focused this study on the activity of the hippocampal network including sharp waves (SWs), carbachol-induced theta oscillations, and interictal-like spikes. We compare simultaneous LFP and optical GCaMP6f fluorescent recordings in Thy1-GCaMP6f mouse hippocampal slices. We observe that SWs produce a clear population GCaMP6f signal above noise with an average magnitude of 0.3% Δ*F*/*F*. This population signal is highly correlated with the LFP, albeit with a delay of 40.3 ms (SD 10.8 ms). The population GCaMP6f signal follows the LFP evoked by 20 Hz stimulation with high fidelity, while electrically evoked oscillations up to 40 Hz were detectable with reduced amplitude. GCaMP6f and LFP signals showed a large amplitude discrepancy. The amplitude of GCaMP6f fluorescence increased by a factor of 28.9 (SD 13.5) between spontaneous SWs and carbachol-induced theta bursts, while the LFP amplitude increased by a factor of 2.4 (SD 1.0). Our results suggest that GCaMP6f is a useful tool for applications commonly considered beyond the scope of genetically encoded calcium indicators. In particular, population GCaMP6f signals are sensitive enough for detecting synchronous network events with sparse firing and sub-threshold activity, as well as asynchronous events with only a nominal LFP. In addition, population GCaMP6f signals are fast enough for monitoring theta and beta oscillations (<25 Hz). Faster calcium indicators (e.g., GCaMP7) will further improve the frequency response for the detection of gamma band oscillations. The advantage of population optical over LFP recordings are that they are non-contact and free from stimulation artifacts. These features may be particularly useful for high-throughput recordings and applications sensitive to stimulus artifact, such as monitoring responses during continuous stimulation.

## Introduction

Fluorescent calcium signals are widely used for monitoring neuronal activity in the brain thanks to the availability of genetically encoded indicators. GCaMP6f is among the best calcium indicators to date, with high sensitivity, high fluorescent yield and relatively fast response time (Chen et al., 2013). There are two principal applications of GCaMP6f: to visualize somatic calcium transients due to bursts of action potentials (Svoboda et al., 1997; Chen et al., 2013; Muto et al., 2013; Dana et al., 2014), and monitoring population activity via calcium fluorometry (Cui et al., 2013; Kupferschmidt et al., 2017). Two important issues related to the measurement of population activity remain less explored: (1) Is GCaMP6f sensitive enough for detecting population events with sparse neuronal firing and sub-threshold synaptic activity? (2) Is GCaMP6f fast enough for detecting physiologically relevant network oscillations?

For the sensitivity issue, we ask whether the population GCaMP6f signal is comparable to local field potential (LFP) recordings, i.e., capable of detecting network activity in which only a small fraction of neurons fire action potentials, while the majority of neurons only have subthreshold potentials. This is of concern as GCaMP fluorescence appears to primarily reflect supra-threshold somatic Ca^2+^ influx (Helmchen and Tank, 2015), together with intracellular calcium induced by action potentials (Grienberger and Konnerth, 2012). In contrast, the principal source of the LFP may rather reflect subthreshold dendritic synaptic currents (Helmchen and Tank, 2015). However, while the Ca^2+^ signals from individual synapses are small, a population signal from the integration of a large number of dendrites in the neuropil might become detectable, given that the sensitivity and fluorescent yield of GCaMP6f are both excellent.

Concerning the frequency response, the time course of somatic calcium transients is about 1 s (Chen et al., 2013; Dana et al., 2014; Miyawaki et al., 2014), which is too slow for detecting most network oscillations. In a recent report (Xing and Wu, 2018), 2 Hz but not 10 Hz GCaMP signals were detectable. However, the rise time of calcium transients is much faster than 1 s and may be able to follow fast oscillations. Measurements with the organic calcium indicator magnesium green showed <1 ms rise time (Regehr, 2000). When action potentials occur, the duration of intracellular calcium transients are long and dependent on intracellular buffering and clearance (Neher, 2013; Helmchen and Tank, 2015). In contrast, during a network oscillation with low calcium influx on average in the population, we argue that the time course will be more related to faster rise time than the slower decay time, and in particular the response time of the calcium indicator, or about 40 ms for GCaMP6f (Chen et al., 2013), as the onset time of the channels and the rate of calcium influx would be only a few milliseconds (Demuro and Parker, 2004; Shuai and Parker, 2005). When only a small fraction of neurons fire action potentials in the population, the majority of neurons should have a rate of calcium influx related to the opening probability of low voltage activated channels (LVAs) (Reviewed by Catterall, 2000; Grienberger and Konnerth, 2012). The opening probability of the LVA channels should be correlated to the fluctuation of membrane potential. In theory, recording fast oscillations in neuronal populations with calcium indicators is possible during sparse firing, when only a small fraction of neurons fire action potentials, most neurons have subthreshold membrane potential fluctuations, and the ability of intracellular calcium buffering/clearance is much greater than the calcium influx rate. The response time of GCaMP6f (∼40 ms) could in theory permit the detection of oscillations up to 25 Hz.

Hippocampal sharp waves (SWs) are spontaneous network events in which a small fraction of neurons fire action potentials (Ylinen et al., 1995; Csicsvari et al., 1999a,b; Mizuseki and Buzsaki, 2013) while the majority of neurons receive subthreshold excitatory and inhibitory synaptic input (Hajos et al., 2013). The summation of excitatory and inhibitory post-synaptic potentials (EPSPs/IPSPs) generates a clear voltage source/sink pair in LFP recordings (Maier et al., 2009), reviewed by Buzsaki (2015). In this report we test if spontaneous SWs can be seen in the GCaMP signal, events with sparse firing and sub-threshold synaptic activity. We speculate that the population summation of calcium influx during these events is detectable in GCaMP6f optical recordings.

We compared simultaneously recorded LFP and fluorescent GCaMP6f signals in Thy1-GCaMP6f mouse hippocampal slices during SWs, interictal spikes and carbachol-induced theta oscillations. Population activation by electric stimulation was also used to test the frequency response characteristics of GCaMP6f population signals.

We observed that SWs can be clearly detected optically in the population GCaMP6f signal. The GCaMP signals were highly correlated with LFP-detected events with a delay of 40.3 ms (SD 10.8 ms). The GCaMP signal followed evoked network activity below 20 Hz with high fidelity, while activity up to 40 Hz were still detectable with reduced amplitude. The population GCaMP6f and LFP signals showed a large amplitude discrepancy. The amplitude of GCaMP6f fluorescence increased by a factor of 28.9 (SD 13.5) between spontaneous SWs and carbachol-induced theta bursts, while the LFP amplitude increased by a factor of 2.4 (SD 1.0).

Our results suggest that the population GCaMP6f signal has a sensitivity comparable to that of the LFP and may be even more sensitive than the LFP for detecting network events with low amplitude but disproportionally large GCaMP6f fluorescence, likely arising from asynchronous activity. We also found that the population GCaMP6f signal is fast enough for monitoring theta (4–7 Hz) and beta (14–25 Hz) oscillations in slice, and the detection limit can be as high as 40 Hz. These results suggest that optical recordings of population GCaMP6f signals may be useful for detecting network activity complementary to LFP recordings. They may have particular utility in situations where the confound of electrode disruption and stimulus artifacts need to be minimized. In addition, they have the power to monitor activity in multiple distinct anatomical sites concurrently. Our results also have implications for the interpretation of *in vivo* data obtained with the increasingly widespread use of GCaMP based photometry (Cui et al., 2013; Kupferschmidt et al., 2017).

## Materials and Methods

### Slice Preparation

P21–P33 male and female C57BL/6J-Tg (Thy1-GCaMP6f) GP5.5 Dkim/J. mice (Jax 024276) mice were used to prepare paired hippocampal hemi-slices in accordance with a protocol approved by the Institutional Animal Care and Use Committee at Georgetown University Medical Center. Following deep isoflurane anesthesia, animals were rapidly decapitated. The whole brain was subsequently removed and chilled in iced (0°C) sucrose-based artificial cerebrospinal fluid (sACSF) containing (in mM) 252 sucrose; 3 KCl; 2 CaCl_2_; 2 MgSO_4_; 1.25 NaH_2_PO_4_; 26 NaHCO_3_; 10 dextrose; bubbled with 95% O_2_, 5% CO_2_. Hippocampal slices (480 μm thick) were cut in horizontal sections from dorsal to ventral brain with a vibratome (Leica, VT1000S). Slices were incubated in ACSF for at least 2 h before each experiment. ACSF used for maintenance and recording contained (in mM) 132 NaCl; 3 KCl; 2 CaCl_2_; 2 MgSO_4_; 1.25 NaH_2_PO_4_; 26 NaHCO_3_; 10 dextrose; bubbled with 95% O_2_, 5% CO_2_ at 26°C.

### Local Field Potential (LFP) Recording

Local Field Potential (LFP) recordings were done in a submerged chamber, and slices were placed on a mesh that allowed perfusion on both sides at a high flow rate (10–30 ml/min) (Hajos and Mody, 2009; Maier et al., 2009). All recordings were done with low resistance glass microelectrodes (∼150 kΩ tip resistance). The electrodes were pulled with a Sutter P87 puller with six controlled pulls and filled with 0.5 M NaCl in 1% agar, which prevents leakage of the electrode solution that could potentially alter the tissue surrounding the electrode tip. The recording electrode was placed in CA1 stratum *pyramidale*, where SWs have large amplitudes (Maier et al., 2009) in healthy slices.

### GCaMP Fluorescent Recording

The GCaMP signals were recorded by a 464-channel photodiode array (WuTech Instruments). The two-stage amplifier circuits in the diode array subtract the resting light intensity and amplify the small optical signals 100 times before digitization. This achieves a 21-bit effective dynamic range to fully digitize a signal of ∼0.5% Δ*F*/*F*. [For a recent review of the two-stage imaging system, see Liang et al. (2015)].

The Δ*F*/*F* is defined as (*F_x_*−*F*_0_)/*F*_0_, where *F_x_* is the signal trace from each detector and *F*_0_ is the baseline fluorescent intensity. The signals were digitized at 1,616 frames/s. In some experiments, only the center of the field of view was sampled at 3,000 Hz, in order to preserve the high frequency components in the signal. The LFP and stimulation signals were sampled and digitized concurrently with the VSD signals. Optical imaging was performed on an upright microscope (Olympus BX51 WI) with an epi-illumination arrangement: excitation light (470 nm LED, ThorLabs) passes a GFP filter cube (Chroma, excitation 425–475 nm, dichroic mirror 480 and emission filter 485 long pass). The GCaMP signals were imaged at two spatial resolutions: A 20× objective (0.95 NA, Olympus) permitted the concurrent imaging of more localized cellular activity and population signals from the same tissue, and a 10× objective (0.30 NA, AMscopes) allowed for imaging all hippocampal subfields in the same field of view. The aperture of the diode array was 19 mm in diameter; containing a hexagonal arrangement of 464 optic fibers. The diameter of the fiber was 750 μm. Each detector on the array (pixel) collected florescent signals from an area of 37.5 μm in diameter with the 20× objective, and about 75 μm in diameter with the 10× objective. The total beam power of the LED was ∼350 mW at 1A. The fluorescent intensity on each detector was about 20,000 photoelectrons/ms for the 20× objective. Illumination intensity at the sample was <20 mW/mm^2^.

### Voltage-Sensitive Dye (VSD) Imaging

Voltage-sensitive dye imaging was used to validate the response time of the GCaMP signal (Figure 3). In three slices, VSD and GCaMP signals are from the same tissue and imaged by the same diode array. The slices were stained by an oxonol dye, NK3630 (Nippon Kankoh-Shikiso Kenkyusho Co., Ltd., Japan), as an indicator of transmembrane potential. Slices were stained with 5–10 μg/ml of the dye dissolved in ACSF for 120 min (26°C). During staining, the ACSF was circulated and bubbled with 95% O_2_, 5% CO_2_. After staining, the slices were transferred back to the incubation chamber for at least 1 h before each experiment. NK3630 binds to the external surface of the membrane of all cells and reports their membrane potential change [for a recent review of the diode array and NK3630, see Liang et al. (2015)]. The absorption spectrum of the dye shifts linearly with the changes in the membrane potential (Ross et al., 1977). The VSD signal in this report is the change in absorption of light with a 705 nm wavelength. The detectable signals are a change in light intensity that is roughly 0.01–0.1% of the resting light intensity. Staining with this dye does not cause noticeable changes in spontaneous or evoked neuronal activity (Jin et al., 2002; Huang et al., 2004), and stained slices maintain viability for up to 24 h. In 705 nm recording light, NK3630 molecules do not generate fluorescence, so no noticeable phototoxicity is detected (Jin et al., 2002).

The VSD signals were recorded by the same diode array. With a transillumination arrangement, neurons through the whole thickness of the slice (480 μm) contribute relatively equally to the VSD signal. A tungsten filament lamp was used for illumination and a 705/10 nm interference filter (Chroma) was placed in the illumination path during optical recording.

During imaging experiments, the slice was continuously perfused in a submersion chamber with ACSF (same as the incubation solution) at 26°C and at a rate of more than 20 mL/min. Intermittent VSD imaging trials were performed, with 2–3 min intervals between trials.

### Stimulation

Stimulation to the CA3 area was provided with a concentric metal electrode (FHC CBDSE 75). Stimulation pulse was 0.1 ms wide generated by a Master 8 stimulator (AMPI). The stimulation current was 20–100 μA generated by an isolator (AMPI).

### Data Analysis

**FIGURE 1:**
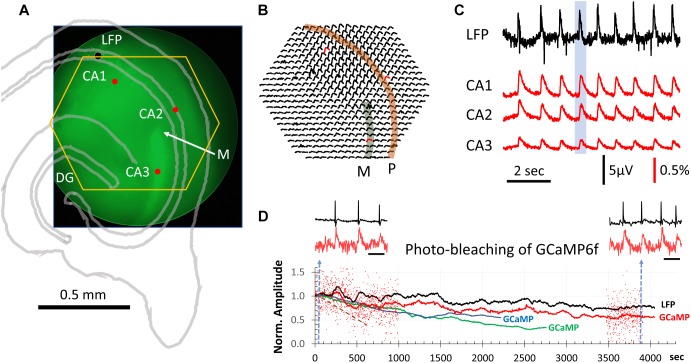
GCaMP population signals of SWs across hippocampal subfields. **(A)** Slice positioning over the diode array. The end of mossy fibers (M) and lack of GCaMP6f expression in CA2 was used to identify the three hippocampal subfields CA1-3. The yellow hexagon marks the field of view of the diode array. The black dot marks the location of the LFP electrode. These structures were also outlined with an image of transmitted light (gray lines). **(B)** Δ*F*/*F* from all 464 diodes during a SW event. This SW was one of the 9 occurring during a 9-s recording sweep (blue box in **C**). Note that GCaMP signals of SWs were seen over a large area across CA1, CA2, and CA3. Str. *pyramidale* (P, orange band) and mossy fiber bundle (M. f∖green band) are identified overlaying the signals. **(C)** LFP signals were simultaneously recorded with the GCaMP signals (both sampled at 1,616 Hz). Signals from three detectors in CA1, CA2 and CA3 [red dot/traces in panels **(A,B)**] plotted together with the LFP recording (filtered 0.1–30 Hz). The amplitude of SWs in the GCaMP signal were on average 0.3% Δ*F*/*F* with a signal-to-noise >10. From 11 slices we recorded ∼6,500 SW events optically, all with a clear one-to-one correspondence between LFP and optical GCaMP signals. **(D)** Decline of optical signals over long recording periods due to photobleaching. Red dots mark the relative amplitude of individual SWs from one slice recording. For clarity, events are only shown for the first 1,000 and last 500 s. The relative Δ*F*/*F* amplitude is normalized to the average amplitude of the first 100 events at the beginning of light exposure. Black and red traces are averages of the LFP and GCaMP signals, respectively, in a sliding 100-event window. Blue and green traces are GCaMP signals from two additional animals. Brown broken line: another slice with exposure at 6 times the light intensity for 660 s. Left and right insets: LFP and GCaMP signals from one slice before and after 4300 s of continuous light exposure. Blue broken line in panel **(D)** marks the sample time of the two traces. Note that amplitude reduction due to photobleaching is not obvious in individual SWs, as spontaneous SWs have a large variation in amplitude. Scale bar for inset = 1 s.

Digital filters were applied offline. To automatically detect the amplitude and peak time of the SW events in fluorescent signals (e.g., Figure 1D), we first digitally filtered the simultaneously recorded LFP signals between 1–30 Hz, then a threshold was set manually above the baseline noise to identify the majority of SW events in the LFP. Using a window between −50 and 100 ms of the LFP peak, the peak of the fluorescent signal was identified as the SW peak. Custom programs were written in MATLAB and Labview for digital filtering, threshold detection, and determining the amplitude and frequency distributions. For figure preparation, various bandpass ranges were chosen for the LFP and GCaMP signals to minimize filtering whenever possible. These specific ranges have been identified in figure legends.

In experiments with high frequency stimulation, in which the amplitude of the response was too small for quantification (Figure 3E), we used the root mean square (RMS) power in place of the amplitude. The RMS was calculated with the following equation:

$$X_{\mathrm{RMS}}=\sqrt{\frac{1}{N}\sum_{n=1}^{N}|X_{n}{|}^{2}}$$

where *X_n_* is the signal amplitude at the sample point *n*, *X_RMS_* is the RMS power in a period of *N* sampling points.

Statistics were conducted in Graphpad Prism 8.0. To compare differences in means we first checked normality and lognormality of data with Shapiro-Wilk tests. Differences in means of two groups were assessed by unpaired *t*-tests for parametric distributions, and Mann-Whitney for non-parametric distributions. For more than two groups we compared means via 1-way ANOVA with the Tukey multiple comparisons correction, or Kruskal-Wallis with Dunn’s multiple comparisons test as appropriate. Error bars displayed are either SD or SEM, as indicated in figure legend. ^∗^*p* < 0.05, ^∗∗^*p* < 0.01, ^∗∗∗^*p* < 0.001, ^∗∗∗∗^*p* < 0.0001.

## Results

### Spontaneous SWs Are Detectable in the Population GCaMP Signal

Spontaneous SWs reliably occur in hippocampal slices as reported by our previous papers and other groups (Kubota et al., 2003; Maier et al., 2003; Colgin et al., 2004; Behrens et al., 2005; Jiang et al., 2018; Sun et al., 2018). Our first goal was to test if SWs can be detected in the GCaMP6f signal in hippocampal slices from Thy1-GCaMP6f mice.

To test this, we positioned the slices with the end of the supra-pyramidal mossy fiber bundle at the center of the field of view of the diode array (Figure 1A), as this marks the boundary between CA3 and CA2 (Blackstad et al., 1970; Gaarskjaer, 1978). In Thy1-GCaMP6f mice the mossy fibers showed bright green fluorescence, due to the high expression in granule cells, providing a landmark for the outside limit of CA3 (“M” in Figure 1A). CA2 could also be clearly identified as a darker region devoid of fluorescent cell bodies. In this way, CA3, CA2, and CA1 could be clearly delineated. By positioning the end of the mossy fiber bundle at the center of the field of view, the 464 detectors on the diode array covered a large area including CA3, CA2, and CA1 (yellow hexagon in Figure 1A).

We observed one-to-one correlations between SW events detected in the LFP and GCaMP signals. In contrast to localized cellular calcium transients (Miyawaki et al., 2014), the population SW signals were reliably seen over a large area of hippocampal tissue spanning CA3, CA2, and CA1 (Figure 1B,C). Under a 20× objective, each optical detector received light from an area of 37.5 μm in diameter, so that the population signals we refer to in this report are a summation of the Δ*F*/*F* from both somatic and dendritic areas for a number of neurons under each optical detector.

Sharp wave peaks in the GCaMP signal were visible across trials, with a range in Δ*F*/*F* of 0.1–1.0% (Figure 1C). The signal-to-noise ratio was >10, allowing clearly distinguishable events above noise. LFP signals were simultaneously recorded with the GCaMP signals, both sampled at 1,616 Hz. From 11 slices we recorded 6,500 SW events optically, all with a one-to-one correspondence between LFP and optical recording of the GCaMP signals, with an average Δ*F*/*F*∼0.3%.

### Signal Polarity of SWs Across Hippocampal Layers

A notable difference between LFP and GCaMP recordings of SWs is the signal polarity in different laminar areas. LFP signals from str. *oriens* and str. *radiatum* have opposite polarities (Maier et al., 2009), as they form a current source-sink pair around str. *pyramidale* (Johsdon and Wu, 1994). This polarity reversal is obviously not observable in the Δ*F*/*F* (Figure 1B). GCaMP signals from soma (str. *pyramidale*) and neuropil (polymorphic or molecular layers) have the same polarity (increased Δ*F*/*F* at SW onset), suggesting that the calcium signals increase irrespective of current flow direction in the population.

### Photo-Bleaching Limits Long Recording Times

Continuous exposure to light while recording causes bleaching of the GCaMP fluorescent protein. We did observe a gradual reduction in SW amplitude with exposure time. To test the limits of optical recordings of SWs, we recorded continuously for over an hour (4,300 s). In a representative experiment (Figure 1D), the excitation light intensity was reduced to 1/4 of the intensity used in Figure 1C. Under this light intensity the SWs were still reliably detected, while the dark noise (noise in the electronics) became larger (Figure 1D, inset red traces). After long exposure, the amplitude of SWs in the optical signal reduced but were still distinguishable from noise. Because amplitude of spontaneous SWs varies over a large range (Figure 1D, red dots), amplitude reduction by photo-bleaching was often difficult to see from individual SW events. However, when the average of 100 SWs were plotted (Figure 1D, red curve), a clear trend of amplitude reduction in the optical signal was seen, compared to the simultaneously recorded LFP amplitude (Figure 1D, black curve). Similar long-duration recordings were performed in three slices from three animals (Figure 1D, blue and green traces). In these experiments the illumination intensity was kept constant, revealing slightly different rates of amplitude reduction. From these results we determined that reliable optical recordings were possible for at least 1,500 s of recording time with continuous illumination, equivalent to 100 15 s trials or 1,000–2,000 spontaneous SWs. At the illumination intensity 6 times greater than in Figure 1D (red, green, and blue), the bleaching rate was much faster, with the amplitude reduced to 50% in 660 s of exposure (Figure 1D, brown dashed line).

### Time Delay Between GCaMP and VSD Signals

**FIGURE 2:**
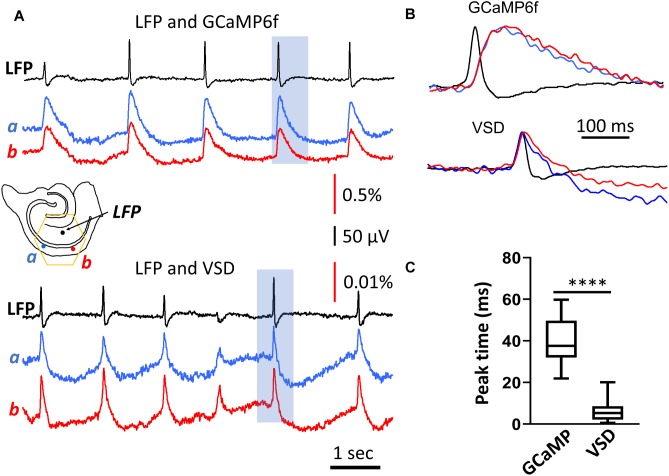
Rise time of the population GCaMP6f signal. **(A)** LFP and GCaMP/VSD recordings of SWs in the same tissue. LFP (black trace, filtered 0.2–50 Hz) were highly correlated with events detected optically at two different CA1 locations either with GCaMP6f (blue and red traces, top, filtered 0.2–50 Hz) or VSD (blue and red traces, top, filtered 0.2–50 Hz). Note the different y-scale for GCaMP and VSD traces, GCaMP events were about 50 times larger and slower. Black arrows mark the SWs displayed in expanded time scale in panel **(B)** GCaMP signals showed a marked delay compared to the LFP, which was not observed in the VSD signals from the same tissue. **(C)** Box and whisker plots of the peak delay time between LFP and optical signals. As expected, GCaMP signals showed a longer delay time of 40.3 ms (SD 10.8 ms, *n* = 84 SWs, in two slices from two animals) compared to VSD delay time of 5.7 ms (SD 4.1 ms, *n* = 82 SWs from same slices), a significant difference of *p* < 0.0001 (Mann-Whitney).

GCaMP signals showed a significant delay compared to LFPs (Figure 2A,B, top traces). The peak of the population GCaMP6f signal lagged behind the peak of the LFP signal by 40.3 ms on average (SD 10.8 ms, *n* = 84 SWs, in two slices from two animals) (Figure 2C). The delay time from two recording locations (Figure 2A, location a, b, ∼1.2 mm apart along the CA1 str. *pyramidale*) was similar (Figure 2B, top traces), suggesting that the delay was not caused by spreading of the SW along the CA1 zone.

The delay time of the GCaMP6f population signal was verified with voltage-sensitive dye recordings. In three animals we stained the slices with the voltage sensitive dye NK3630. The dye staining did not affect the spontaneous SW rate of occurrence or amplitude. The dye absorption signal measured at 705 nm emission wavelength was able to detect SWs. The VSD signal associated with a SW event was manifested by an increase in absorption at 705 nm, in accordance with the previously established nature of voltage imaging with absorption dyes, where dye molecules bind to the neuronal membrane with depolarized membrane potential (Ross et al., 1977). The VSD signal of SWs was fast with the peak correlating well with the LFP (Figure 2A,B, bottom traces). The VSD signal showed an insignificant delay to the LFP, 5.7 ms (SD 4.1 ms, *n* = 82 SWs, in two slices from two animals), demonstrating a good correlation between the population summation of membrane potential and LFP signal during SW events.

The GCaMP and VSD signals in Figure 2 were measured from the same tissue in different recording trials. In the same tissue the VSD and the GCaMP signals were independent as the 705 nm light did not excite the GCaMP6f proteins. The GCaMP signals were measured at 500–530 nm (excited by 470 nm), and there is no significant contribution of VSDs at this wavelength.

A wavelength independent “intrinsic” optical signal was also seen at 705 nm in the VSD measurements. The intrinsic signal was slower and with a reversed polarity compared to the VSD signals at the 705 nm (Jin et al., 2002). The downward deflection in the VSD (Figure 2A,B bottom traces) were associated with this intrinsic optical signal.

Comparing with the VSD signals, the time delay in population GCaMP6f signals was likely dictated by the response of the GCaMP protein. The delay times in our population measurements were comparable to the delay times obtained using intracellular calcium measurements; a 40 ms rise time (Chen et al., 2013).

### Population GCaMP Signal Can Detect 20–40 Hz Evoked Activity

**FIGURE 3:**
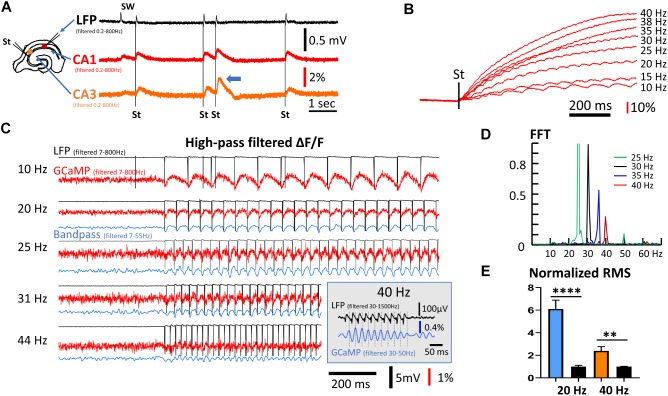
Frequency response of population GCaMP signals. **(A)** GCaMP signals in response to mild electric stimuli. Left: The arrangement for the stimulation and recording. Stimuli were applied to CA3 and response was recorded in both CA1 and CA3. The stimulation intensity was adjusted so that the evoked response had an amplitude similar to the SW amplitude in the LFP signals. Note that double stimuli induced larger responses to the second shock, even with long inter-stimulus intervals of 500 ms (blue arrow head). **(B)** High frequency stimuli caused a ramp accumulation of GCaMP signals. Note that the ramp signal was much larger and slower than the response to individual stimuli. **(C)** Evoked GCaMP signal to high frequency stimuli. A high frequency component can be seen in wide-band filtered signals (red traces, 7–800 Hz). Narrow band filtered (blue traces, 7–55 Hz) improved signal-to-noise ratio. (Inset) In a follow-up experiment, 10 mild stimuli (1.4× threshold) at 40 Hz was given to CA3. A narrow bandpass filter (30–50 Hz) of the CA1 GCaMP signals reveals a clear one-to-one correlation between individual stimulus pulses (filtered 30–1500 Hz) and the GCaMP response. **(D)** Power spectrum of the GCaMP signals, normalized to the power at 30 Hz. The signal power reduced to ∼50% at 35 Hz and ∼25% at 40 Hz. Green peak at the 50 Hz is the 2nd harmonic of the 25 Hz peak. High frequency, weak stimuli experiments were done on three slices from three animals. Data in panels **(A,D)** are from different slices receiving stimuli of slightly different frequency. **(E)** RMS power quickly reduced in high frequency. The RMS power during 20 or 40 Hz stimulations was normalized to the RMS noise in the same trial when there was no stimulation. Blue and left black bars, narrow band-pass filtered between 5–30 Hz, *n* = 8 trials, three slices from three animals, ^∗∗∗∗^*p* < 0.0001 (unpaired *t*-test). Orange and right black bars, narrow band-pass filtered between 25–50 Hz, *n* = 9 trials, three slices from three animals, ^∗∗^*p* = 0.0022 (unpaired *t*-test). Error bars indicate SEM.

If individual calcium transients are far from saturation, the measured rise time of ∼40 ms of the population GCaMP6f optical signal should in theory allow following up to 20 Hz of network oscillations in the tissue. It may also be possible to detect higher frequency signals at attenuated amplitude. To test the frequency response of GCaMP6f population signals, we applied electrical stimulation to CA3 and measured the evoked population response in CA1. The stimulation intensity was low, adjusted to produce evoked LFP responses within the same amplitude range as spontaneous SWs in the same tissue (Figure 3A). Evoked GCaMP signals were observed in the CA3 and CA1 areas (Figure 3A, red and orange traces). When two stimuli were delivered close in time, the response to the second stimulus was larger (Figure 3A, arrowhead), suggesting paired-pulse facilitation through buildup of calcium is detectable by population GCaMP signals.

With a train of stimuli of identical intensity, the GCaMP signals summated, forming a much larger rising ramp than the response to individual stimuli (Figure 3B). The Δ*F*/*F* amplitude evoked by individual stimuli were on average 0.1%, while the ramp signal from continuous 40 Hz stimulation was ∼100% (comparing Figure 3B,C). The ramp rise time was faster with higher stimulation frequency with the same intensity (Figure 3B). The large ramp signal suggests an accumulation of calcium. The time course of these long ramps might be caused by the slow clearance of intracellular calcium, while the individual responses may reflect fast calcium influx.

As the rise time of the long ramp signal was much slower than the rise time of individual responses, the ramp signal could be removed by a digital high-pass filter (7–800 Hz) (Figure 3C, red traces). The one-to-one relationship between evoked stimulation and GCaMP signal was maintained up to a frequency of 40 Hz. Responses to stimulation <30 Hz were clearly seen in high-pass filtered signals, while frequencies between 30 and 44 Hz were distinguishable by further band-pass filtering between 7 and 55 Hz (Figure 3C, blue traces). One-to-one correlations between stimulus and GCaMP signals were clearly seen at 31 Hz but not at 44 Hz. In a follow-up experiment, 10 mild stimuli (1.4× threshold) at 40 Hz was given to CA3, and a narrow bandpass filter (30–50 Hz) was used on the CA1 GCaMP signals. Under this condition a clear one-to-one correlation between individual stimulus pulses and the GCaMP response was identified (Figure 3C, inset). Response to the 40 Hz stimuli was further verified with a fast Fourier transform (FFT) (Figure 3D). While the 40 Hz FFT peak was much smaller compared to that of lower frequencies, the peak was clearly distinguishable from background noise. The frequency peaks show about a 50% reduction between 35 and 40 Hz. RMS power was calculated from data collected from three animals receiving 20 and 40 Hz stimulation (Figure 3E), demonstrating that the 40 Hz GCaMP6f signal was significantly higher than the RMS power of background noise (*p* = 0.0022, unpaired *t*-test, *n* = 9 trials from three slices from three mice).

### Amplitude Discrepancy Between GCaMP and LFP Signals

**FIGURE 4:**
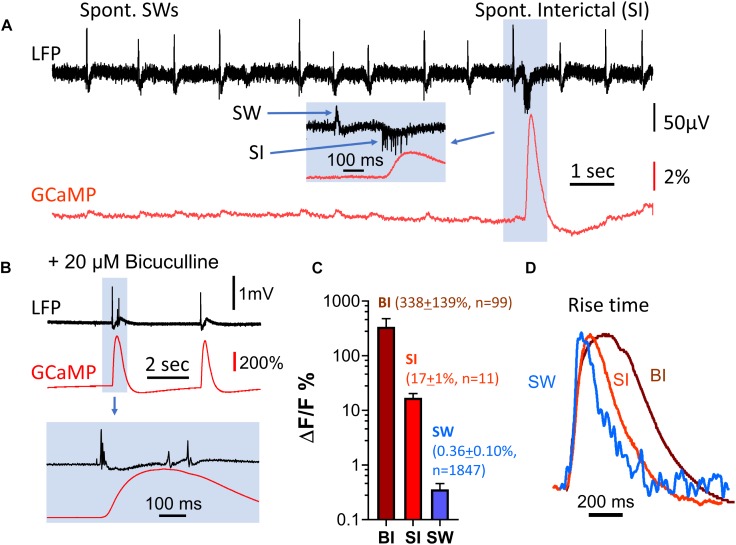
Amplitude discrepancy between LFP and GCaMP signals during interictal-like events. **(A)** Spontaneous interictal events (blue box) rarely occur in healthy slices, and are associated with a low LFP peak amplitude but disproportionally large GCaMP fluorescence. The GCaMP signal was measured from the CA1 area, in a region of interest approximately 0.1 mm in diameter surrounding the location of the LFP electrode. *Inset (blue box)*: expanded time scale of the event, showing cellular spiking and the LFP peak of the interictal event. **(B)** Interictal-like events induced by bath supply of 20 μM bicuculline. Bottom traces display event highlighted in blue box above on expanded time scale. **(C)** Comparison of GCaMP6f Δ*F*/*F* amplitude for three types of events. BI: bicuculline-induced interictal-like spike, average Δ*F*/*F* = 338 (SD 139%, *n* = 99 events from four slices from four animals). SI: spontaneous interictal event, average Δ*F*/*F* = 17% (SD 1%, *n* = 11 events from one slice from one animal), SW: sharp wave, average Δ*F*/*F* = 0.36% (SD 0.10%, *n* = 1847 events from the same slice with SIs). Error bars indicate SD. **(D)** Rise time of sharp wave (SW), spontaneous interictal events (SI) and bicuculline-induced interictal-like spikes (BI). The amplitude of the three events differ by a factor of ∼600 but have similar initial rise times. Note that the decay time of the three events are different. These traces are averages of *n* = 100 SWs from one animal; *n* = 11 SIs from the same animal; *n* = 10 BIs from a different animal.

Exceptionally large GCaMP signals were occasionally observed during population events, while the LFP signals of the same events were relatively small. These spontaneous interictal events had an amplitude of Δ*F*/*F* = 17% (SD 1%, *n* = 11), almost 50 times greater than the amplitude of SWs in the same tissue [Δ*F*/*F* = 0.36% (SD 0.10%, *n* = 1847)] (Figure 4). In contrast, the LFP signals of SWs and interictal spikes had similar amplitudes, but interictal spikes exhibited reversed polarity and increased extracellular spiking (Figure 4A, inset).

Spontaneous interictal events only occurred occasionally in 2 out of 11 slices examined, and their occurrence rate was low. In one characteristic slice, 1,847 SWs were recorded over 36 min, with only 11 spontaneous interictal events detected. To further investigate the discrepancy between amplitudes in the LFP and GCaMP6f signals, we examined induced interictal-like events with the GABA_A_ receptor antagonist bicuculline. 20 μM bicuculline in ACSF was used to induce interictal-like spikes in four slices from four animals (*n* = 99 events, Figure 4B). The amplitudes of GCaMP events associated with bicuculline-induced interictal spikes were almost 1,000 times greater than the amplitudes of the SW events, while the LFP signal had a relative increase ranging from 5 to 20, demonstrating a large discrepancy in relative signal increases between GCaMP and LFP (Figure 4C).

Notably, the rise time, defined as the time for the signal to rise from 10 to 60% of peak, was about 40 ms for all three event types (spont. SW, spont. interictal, bicuculline-induced), despite the large amplitude differences in these events (Figure 4D). This suggests that the onset time of the optical population signal in all three cases is limited by the response time of GCaMP6f.

### Population GCaMP Signal Can Detect Carbachol-Induced Theta Oscillations

**FIGURE 5:**
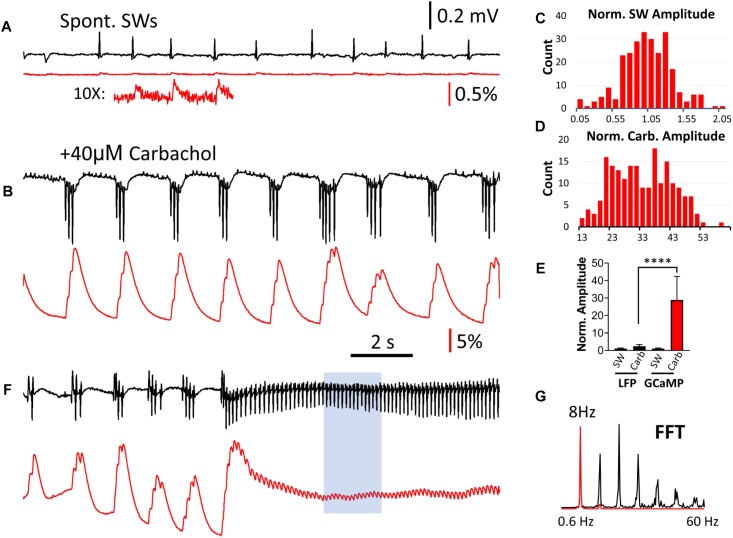
Comparison of LFP and GCaMP signals during carbachol-induced theta oscillations. **(A)** Spontaneous SWs when the slice is bathed in normal ACSF, compared with **(B)** Induced theta bursts recorded from the same tissue after bath administration of 40 μM of the cholinergic agonist carbachol. The LFP and GCaMP signals are drawn on the same scales for panels **(A,B)**. On this amplitude scale the GCaMP SW events are very small, but can be clearly seen with 10× amplification (**A**, Inset). Note that the polarity of the LFP signals were reversed during theta bursts, and a group of three or more spikes in the LFP was merged into a single large peak with GCaMP. **(C)** Amplitude distribution of SWs in the GCaMP signals (*n* = 260 SWs, four slices from four animals), first normalized to the average amplitude from each slice and then pooled together in the distribution chart. **(D)** Amplitude distribution of carbachol-induced theta bursts in GCaMP signals (*n* = 252 bursts, four slices from four animals). The amplitude was normalized to the average SW amplitude in each slice. Note that the majority of bursts were 13–30 times larger than the SW in the same tissue. **(E)** Amplitude discrepancy: When LFP and GCaMP signals during bursts were normalized to the average amplitude of SWs in the same tissue, The increase in GCaMP was 28.9 (SD 13.5, *n* = 252 bursts, four slices from four animals) while the LFP was only 2.37 (SD 1.02, *n* = 252 bursts, four slices from four animals). Significant difference in medians from the Kruskal-Wallis test, *p* < 0.0001 (Dunn’s multiple comparisons correction between LFP and GCaMP during carbachol show *p* < 0.0001). Error bars indicate SD. **(F)** Continuous theta cycles developed with continued carbachol perfusion. These ∼8 Hz oscillations were seen in both LFP and GCaMP signals. **(G)** An FFT of a sub-section of this signal (**F**, blue box) revealed a clear peak at 8 Hz in both the LFP and GCaMP6f signals, as well as higher harmonics.

Carbachol-induced theta oscillations and related population events were next explored to further investigate the ability of GCaMP6f to monitor physiologically relevant hippocampal oscillations. When the perfusant was switched from normal ACSF to one containing 40 μM of the cholinergic agonist carbachol, spontaneous SWs disappeared and short bursts of theta oscillations (4–7 Hz) emerged, as recorded by the LFP electrode (Figure 5A,B). High amplitude GCaMP peaks were observed during these theta bursts, and like interictal-like events, there was a large discrepancy between the change in amplitude in the LFP and GCaMP signals. The GCaMP signal accumulated with successive theta cycles (Figure 5B). While both SW and carbachol-induced bursts had a wide range of amplitude in GCaMP signal (Figure 5C,D), the amplitude of the carbachol-induced burst was on average greater than the amplitude of SW events by a factor of 28.9 (SD 13.5, *n* = 252 bursts, four slices from four animals) (Figure 5E). In contrast, the amplitude of LFP events increased by a factor of only 2.38 (SD 1.02, *n* = 252 bursts, four slices from four animals), further demonstrating the large amplitude discrepancy between LFP and GCaMP signals.

With continued carbachol perfusion, theta oscillations with continuous cycles developed (Figure 5F). These ∼8 Hz oscillations were seen in both LFP and GCaMP signals with one-to-one oscillation. An FFT of a sub-section of this signal (Figure 5F, blue box) revealed a clear peak at 8 Hz in both the LFP and GCaMP6f signals, as well as higher harmonics (Figure 5G).

### Population GCaMP Signal During Transition Period of Elevated and Asynchronous Activity

The large amplitude discrepancy between the LFP and GCaMP6f signals led us to investigate if GCaMP6f can be used to detect population activity insensitive to the LFP. Elevation of asynchronous firing in a neuronal population should generate only a nominal LFP, with asynchronous currents canceling each other out in the volume conductor surrounding the neurons. However, the GCaMP6f signal in this population would be expected to be high, due to the accumulation of calcium from elevated activity. With even higher levels of activity, depolarization block can lead to a cessation of firing and detectable activity in the LFP, yet with GCaMP6f, the elevated calcium that results from this should be readily detectable.

**FIGURE 6:**
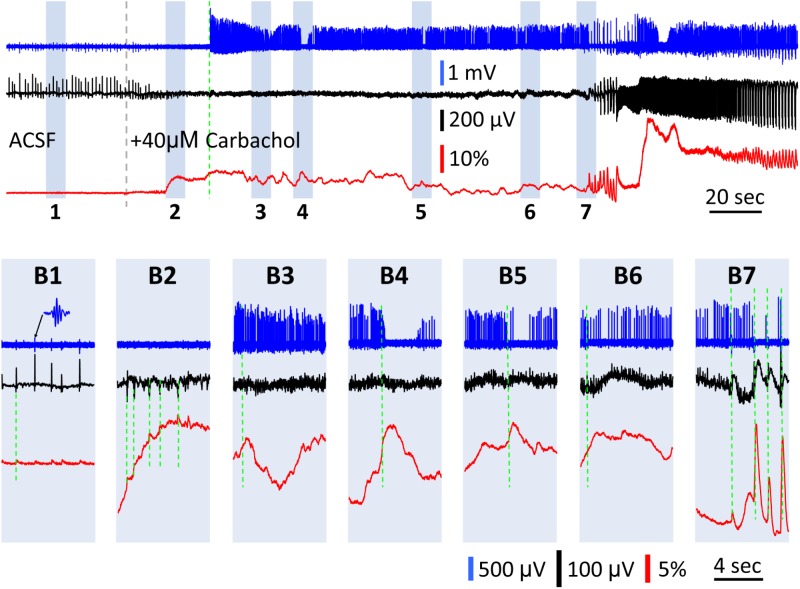
GCaMP signals during the transition between SWs and carbachol induced bursts. **(A)** A 5 min recording of the transition period between spontaneous SWs and carbachol-induced theta oscillations. Blue trace shows the LFP filtered between 60–1,500 Hz (data sampled at 3,000 Hz), to isolate neuronal spikes from nearby cells. Black trace shows the LFP filtered between 0.1–30 Hz to isolate SWs (initial period) and theta bursts (final period). Red trace shows the GCaMP signal filtered between 0.1–30 Hz. Gray broken line marks the onset of 40 μM carbachol perfusion. Green broken line marks the onset of high spiking activity in the filtered blue trace. Particular periods of interest highlighted 1–7 to display on expanded time scale in panels **(B)**. **(B1)** Spontaneous SWs with coincident low amplitude GCaMP peaks (green dashed line) and nested ripple oscillations (inset) before carbachol administration. After carbachol administration, the GCaMP signal had slow and large fluctuations up to Δ*F*/*F* = 30%, or about 40 times the SW signals **(B2–B6)**. **(B2)** A period showing one-to-one correlations between observed peaks in the GCaMP and LFP signals (green dashed lines). **(B3–B6)** Periods showing minimal correlation between LFP and GCaMP fluctuations (comparing black and red traces, green dashed line marks onset of GCaMP peaks). **(B4,B5)** Periods showing anti-correlation between spiking rate and GCaMP fluctuations (comparing blue and red traces, green dashed line marks onset of GCaMP peaks). **(B7)** Onset of theta oscillations, showing large GCaMP peaks, high correlations between LFP and GCaMP, and synchronous bursts of spikes.

There was a transition period between SWs and carbachol-induced bursts, in which the GCaMP signal had large fluctuations while the LFP signal displayed only low amplitude peaks (Figure 6). Upon 40 μM carbachol administration, SWs abruptly stopped (Figure 6A, gray dashed line). A transition period of 3–4 min occurred, during which the GCaMP signal had slow and large fluctuations up to Δ*F*/*F* = 30%, or about 40 times the SW signals (Figure 6B1 vs. Figure 6B2–B6). Meanwhile, the amplitude of the small peaks in the LFP was only 1/3–1/2 of that of the SW (Figure 6B1 vs. Figure 6B2, red traces). These fluctuations only occurred after carbachol was added and SWs stopped (observed in two preparations) and became larger until organized theta bursts emerged (Figure 6B7). At times, one-to-one correlations were observed between peaks in the GCaMP and LFP signals (Figure 6B2). At other times however, these GCaMP signals displayed dynamics not readily apparent in the LFP recording (Figure 6B3–B6). Spikes from nearby neurons were detectable with the LFP electrode in some preparations (Figure 6, blue traces, sampled at 3000 Hz and filtered 60–1500 Hz). Spiking rate increased after carbachol, indicating elevated population activity. Peaks in GCaMP6f were also correlated to sudden drops in the firing frequency of neurons (Figure 6B4,B5). More obvious correlations between reduction in spiking and GCaMP peaks were seen when theta bursts developed (Figure 6B7), potentially from depolarization block or inhibition. Together these observations suggest that in highly excitable and asynchronous environments, GCaMP6f can reveal dynamics not detectable in the LFP, which alone is an incomplete snapshot of population activity.

### Cellular Transients Compared to Population Signals

Finally, we wanted to verify that compared to the population GCaMP6f signal, cellular calcium transients were large, localized to the soma, and with longer duration as previously reported (Miyawaki et al., 2014; Norimoto et al., 2018). In contrast, the population GCaMP signals in the same tissue were expected to be small, distributed over a large area, and with shorter duration.

**FIGURE 7:**
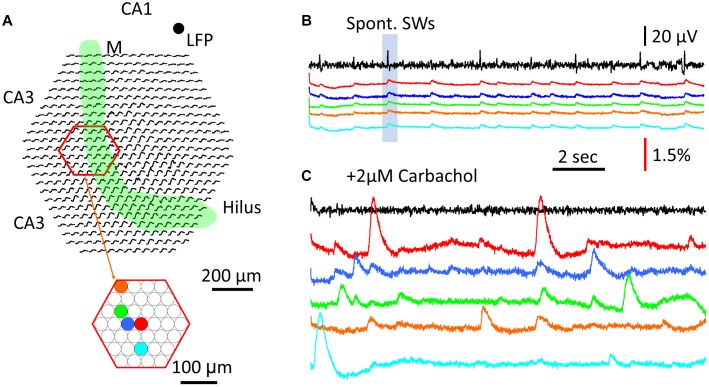
Comparison between cellular transients and population GCaMP signal. **(A)** Schematic diagram of the imaging field. Green band marks the mossy fiber bundle. Red hexagon marks a subset of optical detectors imaging the CA3 pyramidal cell layer. Color dots mark five individual optical detectors; signals on these detectors are displayed in the color traces in panels **(B,C)**. **(B)** Traces of LFP (black) and GCaMP (colors) signals during SW events. LFP was recorded from a location in CA1, marked by the black dot on the top right of panel **(A)**. Color traces were from optical detectors (color dots in panel **A**). Blue box marks the particular SW event with optical recordings from the all detectors (black traces in the imaging field in panel **A**), demonstrating that a large fraction of optical detectors around the mossy fiber bundle show SW signals. **(C)** Cellular Ca^2+^ transients recorded from individual detectors in the CA3 pyramidal layer. Color traces are from the same detectors in panels **(A)**. The signals in panels **(B,C)** were from the same tissue, during normal ACSF and 2 μM carbachol, respectively, a low concentration to promote activity but insufficient to induce theta busts. Note that the cellular Ca^2+^ transients were often localized to individual detectors, and of high amplitude and longer duration than the population SW signals in panels **(A,B)**.

To test this, a region of CA3 str. *pyramidale* was imaged for cellular calcium transients (Figure 7A, red hexagon). Five detectors were chosen to show both SW population signals (Figure 7B) and cellular transients (Figure 7C). In normal ACSF the SW signals were seen in all five detectors (Figure 7B) as well as in most of the detectors throughout the imaging area (SW marked in shaded blue box in Figure 7B displayed over all detectors in Figure 7A). Later, 2 μM carbachol was added to the perfusion solution to promote cellular spiking, a low concentration that was found to be insufficient to induce theta bursts. Under this condition, localized large calcium transients were observed under one or a few detectors, suggesting cellular transients from spiking of individual neurons. Because the diode array had a low spatial resolution, these calcium transients cannot be attributed to individual CA3 neurons. However, these large signals were localized to one or a few detectors; e.g., the signals on neighboring red, blue, and green detectors only showed small crosstalk (Figure 7C), suggesting that the source of the signals was highly localized to the soma or dendrites of distinct CA3 neurons. In contrast, SW signals were distributed over the entire field of view.

The large and localized calcium transients showed a duration of 1–2 s, consistent with cellular calcium transients reported by other groups [e.g., (Dana et al., 2014; Miyawaki et al., 2014)]. The amplitude, spatial distribution, and time course of these local calcium signals all varied, suggesting differing sources.

## Discussion

Our results suggest that Ca^2+^ imaging of population activity can be a useful tool for monitoring activity critical for the consolidation and encoding of memory, e.g., SWs (Figure 1) and theta oscillations (Figure 5). By reliably detecting SW events, these data demonstrate that GCaMP6f is sensitive enough to detect population activity with sparse spiking and sub-threshold activity. With appropriate amplification and filtering of the population signal, we observed that the temporal limit of population Ca^2+^ imaging is close to the response time of the GCaMP6f protein (40 ms), enabling detection of oscillations up to 20 Hz (Figure 3). The range in amplitude for detected population signals with our method spans 3000-fold, from Δ*F*/*F* = 0.1–300% and exhibits a dynamic range different from the accompanying changes to the LFP. In particular, it may be more sensitive than the LFP during highly elevated and asynchronous activity, where the interpretation of the LFP is often ambiguous (Figure 6).

### Temporal Limitations of Population GCaMP6f Signals

The rise time of the population GCaMP6f signal was measured to be 40.3 ± 10.8 ms, which is slower than organic calcium indicators [e.g., Cal-520, Rhof-4, (Lock et al., 2015) reviewed by Grienberger and Konnerth (2012)]. These data indicate that this response time is the major limitation for GCaMP6f to measure fast oscillations. Our results suggest that the speed of GCaMP6f is sufficient for measuring oscillations below 20 Hz, while this can be pushed up to 40 Hz with offline signal processing. A better solution for detecting gamma oscillations would be faster calcium sensitive proteins (Helassa et al., 2015, 2016; Dana et al., 2018).

### Population Signal vs. Cellular Transients

One major question raised by our results is whether the integration of CA1 cellular calcium transient would reproduce the population calcium signal seen during SWs. Cellular calcium imaging during SWs has been performed by the Ikegaya group from a large number of neurons with calcium transients in the CA1 area (Norimoto et al., 2012, 2018; Miyawaki et al., 2014). A careful analysis in these studies (Miyawaki et al., 2014) found that while 79% of neurons displaying calcium transients participated in SW events, each SW event only recruited ∼4% of these neurons. Additionally, each neuron participated in only ∼5% of SW events. Since ∼70% of neurons have calcium transients with and without SWs, it is unclear if integrating the individual cellular calcium transients would generate the population signal we observed in this report [see Figure 4 of Miyawaki et al. (2014)]. A large fraction of uncorrelated cellular transients would only contribute to the background fluorescence.

In contrast, the majority of CA1 neurons receive both excitatory and inhibitory synaptic inputs during every SW event (Hajos et al., 2013). The calcium influx in the presynaptic compartments of both excitatory and inhibitory neurons in the CA1 neuropil would contribute to the population GCaMP6f signal locked to the LFP, given that each CA3 neuron projects to 2/3 of the CA1 area and makes 30,000 to 60,000 excitatory synapses onto CA1 neurons (Li et al., 1994; Wittner et al., 2007). The post-synaptic response of the CA1 neurons might also have activate low threshold calcium channels [reviewed by Catterall (2000)] and contribute to the population calcium signal. The current imaging method cannot distinguish whether the signals are from pre- or post-synaptic calcium influx. Our signals are most likely from both, in addition to the regenerative calcium dynamics in the dendritic tree (dendritic calcium spikes). Further experiments are needed to distinguish the source of the population calcium signal.

Cellular calcium transients reach Δ*F*/*F* = 30–2000% when recorded under a dark background with confocal or two photon microscopes. Under bright field fluorescent imaging the background is no longer dark so the fractional change would be greatly reduced. In our wide-field fluorescent imaging the cellular calcium transients range from 2 to 5 times the population GCaMP signals (Figure 7). The population signal of SWs is a small intensity change over a brighter background (a light flux of ∼10,000 photoelectrons/ms) which would saturate EMCCDs.

### Greater Dynamic Range of Population GCaMP Signal

GCaMP6f and LFP signals showed a striking amplitude discrepancy during population events (Figure 4–6). Spontaneous and bicuculline-induced interictal events, as well as carbachol-induced theta oscillations showed a much greater Ca^2+^ than LFP response. We hypothesize this greater dynamic range in Δ*F*/*F* values compared to the LFP to be due to the population GCaMP6f signal increasing more linearly with increased cellular participation. The LFP will be limited in magnitude due to precise synchrony of voltage-gated and synaptic currents, as well as volume conduction throughout the slice. In contrast, the much slower kinetics of Ca^2+^, and the sensitivity of GCaMP6f to depolarized voltages renders the population GCaMP6f signal more sensitive than the LFP in highly active and/or asynchronous environments.

This high amplitude GCaMP6f signal likely reflects increased population firing rate and not artifact, as we never observed such increases outside of interictal events or carbachol administration. The decay time of GCaMP6f is about 200 ms (Chen et al., 2013), therefore, all calcium transients within the decay period should accumulate and contribute to the population signal. In addition to this, the continued accumulation of intracellular calcium will lengthen the population signal. Individual neurons’ calcium transients last on average 1 s for GCaMP6f [Figure 7, see also (Chen et al., 2013; Dana et al., 2014) as well as measurements with organic calcium indicators (Miyawaki et al., 2014)]. These long duration intracellular calcium transients are limited by calcium buffering/elimination processes (Helmchen and Tank, 2015).

The accumulation of the population GCaMP signal was also clearly seen in the ramp-like signals in Figure 3B, where repetitive stimuli caused a rising ramp, with the ramp slope becoming steeper with higher stimulus frequencies. The ramp signal was much slower and larger compared to the signal induced by individual stimuli. This might partially explain the amplitude discrepancy. In contrast to the population accumulation, on a single cell level, post-synaptic potentials and the GCaMP6 signal time course are much better correlated (Kupferschmidt and Lovinger, 2015).

The accumulation of population GCaMP signals may offer a sensitive indicator for the “spiking density” in the population. Spiking density here refers to an increased firing rate on a temporal scale of ∼100 ms, which is distinct from the more common concept of synchrony, or coincident firing on a millisecond temporal scale. With this definition, high spiking density would not necessarily result in high LFP peaks, as sodium and potassium currents could negate each other if the firing rates between neurons are not closely synchronous.

### Population GCaMP Signals for Detecting Asynchronous Population Activity

During the transition between SW and theta oscillations (Figure 6), the LFP showed only a nominal signal while the population GCaMP signal exhibited large fluctuations. Four observations in Figure 6 suggest that the fluctuations are not noise. First, correlations can be seen between small LFP peaks and GCaMP signals (Figure 6B2), indicating that the fluctuations were not random. Second, the fluctuations only occurred during the transition between the SW and the theta oscillations (Figure 6A), but never during SW states. Third, the fluctuations gradually increased to become large peaks with one-to-one correlation with LFP bursts (Figure 6B7), suggesting the population firing gradually became organized into theta oscillations. Fourth, while the LFP only displayed nominal peaks during the transition, the LFP electrode often picked up spikes from nearby neurons. The high firing rate of nearby neurons suggests an asynchronous state in the population (Figure 6A). In addition, the reduction of cellular firing was weakly correlated to GCaMP6f fluctuations (Figure 6B4,B5,B7).

### SWs vs. Epileptiform Activity

There is an active debate whether *in vitro* SWs are more reflective of epileptiform or other pathological events (Karlocai et al., 2014; Buzsaki, 2015). We demonstrated that the two events have large differences in GCaMP6f characteristics. Spontaneous interictal events, while rare, can happen without changing the bath solution or the excitability of the slices in our preparation (Figure 3A). While the LFP did display altered shapes between the events, the GCaMP6f response was even more highly divergent, indicating that they are different types of events. This provides evidence that *in vitro* SWs are distinct from epileptiform activity.

### Limitations and Advantages of the Diode Device

In contrast to many calcium imaging experiments, we employed a diode array for our measurements. The limiting factor for the sensitivity of the device is the dark noise of the electronics. The intensity of excitation light needs to be high enough so the signal can be distinguished from the dark noise. Bleaching of GCaMP6f fluorescence is a major limitation for the method. In order to achieve >30 min of optical recording time, the excitation light needs to be adjusted as low as possible while maintaining sufficient signal to noise. Under light illumination intensity, the dark noise becomes a major limitation for small signals. When approximately 100 mW of LED light output was used through a 10 × 0.3 NA objective, which delivers <1 mW/cm^2^ onto the tissue, resulting in dark noise about 20% of the SW peaks. Higher excitation power may also get better sensitivity with a trade-off in optical recording time. However, 30 min of light exposure was more than enough for many experiments.

The high dynamic range of the diode array is a main advantage. For detecting 0.1% Δ*F*/*F* on top of 100% background fluorescent light, a 16–20 bit effective dynamic range would be needed. Such high dynamic range is necessary for detecting small population signal with a sensitivity comparable to LFP.

Some of our results are compatible with a recent *in vivo* photometry study (Kupferschmidt et al., 2017), in which high frequency stimuli generated a slow accumulative response and faster individual responses, and epileptiform events displayed high amplitude GCaMP6s signals. We were able to resolve fast signals up to 30 Hz on single trials, and able to record small signals (∼0.1 Δ*F*/*F*) during hippocampal SWs. This is due to the diode array having a higher dynamic range and signal-to-noise ratio than photomultiplier-based devices. Further work is needed to verify if the high signal-to-noise ratio can be achieved under *in vivo* conditions with optical fibers.

## Conclusion

In conclusion, this work demonstrates that population GCaMP signals offer a useful complementary approach to image small and fast population activity, with comparable sensitivity to LFP recordings. A planned future direction is *in vivo* imaging of spontaneously occurred theta (4–8 Hz), alpha/mu (7–13 Hz) and beta (15–20 Hz) oscillations. Faster calcium sensors may allow more robust detection and monitoring of gamma oscillations in the 40 Hz range. GCaMP permits multiple-site no-contact recordings, revealing spatiotemporal dynamics of neuronal oscillations. In addition, optical signals are not disturbed by the artifact of electrical stimulation, suitable for applications requiring simultaneous recording and stimulation, e.g., augmenting EEG oscillations by transcranial repetitive AC or magnetic stimulation [Zhai group 2019, current biology 2019], which currently no other methods can achieve.

## Ethics Statement

This study was carried out in accordance with the laboratory animal welfare guidelines, NIH Office of Laboratory Animal Welfare. The protocol was approved by the Institutional Animal Care and Use Committee of Georgetown University Medical Center.

## Author Contributions

PL, HJ, and JYW conducted the experiments. All authors participated in the data analysis and the composition of the manuscript.

## Conflict of Interest Statement

WuTech Instruments is a company owned by JYW. The diode array used in this research is a gift of WuTech Instruments. The remaining authors declare that the research was conducted in the absence of any commercial or financial relationships that could be construed as a potential conflict of interest.

## References

[B1] Behrens C. J. van den Boom L. P. de Hoz L. Friedman A. Heinemann U. (2005). Induction of sharp wave-ripple complexes in vitro and reorganization of hippocampal networks. *Nat. Neurosci.* 8 1560–1567. 10.1038/nn1571 10.1038/nn1571 16222227

[B2] Blackstad T. W. Brink K. Hem J. Jeune B. (1970). Distribution of hippocampal mossy fibers in the rat. An experimental study with silver impregnation methods. *J. Comp. Neurol.* 138 433–449. 10.1002/cne.901380404 4907846

[B3] Buzsaki G. (2015). Hippocampal sharp wave-ripple: a cognitive biomarker for episodic memory and planning. *Hippocampus* 25 1073–1188. 10.1002/hipo.22488 26135716 PMC4648295

[B4] Catterall W. A. (2000). Structure and regulation of voltage-gated calcium channels. *Annu. Rev. Cell Dev. Biol.* 16 521–555. 10.1146/annurev.cellbio.16.1.521 11031246

[B5] Chen T. W. Wardill T. J. Sun Y. Pulver S. R. Renninger S. L. Baohan A. (2013). Ultrasensitive fluorescent proteins for imaging neuronal activity. *Nature* 499 295–300. 10.1038/nature12354 23868258 PMC3777791

[B6] Colgin L. L. Kubota D. Jia Y. Rex C. S. Lynch G. (2004). Long-term potentiation is impaired in rat hippocampal slices that produce spontaneous sharp waves. *J. Physiol.* 558(Pt 3), 953–961. 10.1113/jphysiol.2004.068080 15194734 PMC1665012

[B7] Csicsvari J. Hirase H. Czurko A. Mamiya A. Buzsaki G. (1999a). Fast network oscillations in the hippocampal CA1 region of the behaving rat. *J. Neurosci.* 19:RC20. 10.1523/JNEUROSCI.19-16-j0001.1999 PMC6782850 10436076

[B8] Csicsvari J. Hirase H. Czurko A. Mamiya A. Buzsaki G. (1999b). Oscillatory coupling of hippocampal pyramidal cells and interneurons in the behaving Rat. *J. Neurosci.* 19 274–287. 10.1523/jneurosci.19-01-00274.1999 9870957 PMC6782375

[B9] Cui G. Jun S. B. Jin X. Pham M. D. Vogel S. S. Lovinger D. M. (2013). Concurrent activation of striatal direct and indirect pathways during action initiation. *Nature* 494 238–242. 10.1038/nature11846 23354054 PMC4039389

[B10] Dana H. Chen T. W. Hu A. Shields B. C. Guo C. Looger L. L. (2014). Thy1-GCaMP6 transgenic mice for neuronal population imaging in vivo. *PLoS One* 9:e108697 10.1371/journal.pone.0108697 PMC4177405 25250714

[B11] Dana H. Sun Y. Mohar B. Hulse B. Hasseman J. P. Tsegaye G. (2018). High-performance GFP-based calcium indicators for imaging activity in neuronal populations and microcompartments. *bioRxiv* [Preprint]. 10.1101/434589 31209382

[B12] Demuro A. Parker I. (2004). Imaging the activity and localization of single voltage-gated Ca(2+) channels by total internal reflection fluorescence microscopy. *Biophys. J.* 86 3250–3259. 10.1016/S0006-3495(04)74373-74378 15111438 PMC1304190

[B13] Gaarskjaer F. B. (1978). Organization of the mossy fiber system of the rat studied in extended hippocampi. II. Experimental analysis of fiber distribution with silver impregnation methods. *J. Comp. Neurol.* 178 73–88. 10.1002/cne.901780105 75894

[B14] Grienberger C. Konnerth A. (2012). Imaging calcium in neurons. *Neuron* 73 862–885. 10.1016/j.neuron.2012.02.011 22405199

[B15] Hajos N. Karlocai M. R. Nemeth B. Ulbert I. Monyer H. Szabo G. (2013). Input-output features of anatomically identified CA3 neurons during hippocampal sharp wave/ripple oscillation in vitro. *J. Neurosci.* 33 11677–11691. 10.1523/JNEUROSCI.5729-12.2013 23843535 PMC3724544

[B16] Hajos N. Mody I. (2009). Establishing a physiological environment for visualized in vitro brain slice recordings by increasing oxygen supply and modifying aCSF content. *J. Neurosci. Methods* 183 107–113. 10.1016/j.jneumeth.2009.06.005 19524611 PMC2753642

[B17] Helassa N. Podor B. Fine A. Torok K. (2016). Design and mechanistic insight into ultrafast calcium indicators for monitoring intracellular calcium dynamics. *Sci. Rep.* 6:38276 10.1038/srep38276 PMC5138832 27922063

[B18] Helassa N. Zhang X. H. Conte I. Scaringi J. Esposito E. Bradley J. (2015). Fast-response calmodulin-based fluorescent indicators reveal rapid intracellular calcium dynamics. *Sci. Rep.* 5:15978 10.1038/srep15978 PMC4630588 26527405

[B19] Helmchen F. Tank D. W. (2015). A single-compartment model of calcium dynamics in nerve terminals and dendrites. *Cold Spring Harb. Protoc.* 2015 155–167. 10.1101/pdb.top085910 25646507

[B20] Huang X. Troy W. C. Yang Q. Ma H. Laing C. R. Schiff S. J. (2004). Spiral waves in disinhibited mammalian neocortex. *J. Neurosci.* 24 9897–9902. 10.1523/jneurosci.2705-04.2004 15525774 PMC4413915

[B21] Jiang H. Liu S. Geng X. Caccavano A. Conant K. Vicini S. (2018). Pacing hippocampal sharp-wave ripples with weak electric stimulation. *Front. Neurosci.* 12:164 10.3389/fnins.2018.00164 PMC5862867 29599704

[B22] Jin W. Zhang R. J. Wu J. Y. (2002). Voltage-sensitive dye imaging of population neuronal activity in cortical tissue. *J. Neurosci. Methods* 115 13–27. 10.1016/s0165-0270(01)00511-8 11897360

[B23] Johsdon D. Wu M.-S. (1994). *Foundations of Cellular Neurophysiology.* Cambridge, MA: MIT Press.

[B24] Karlocai M. R. Kohus Z. Kali S. Ulbert I. Szabo G. Mate Z. (2014). Physiological sharp wave-ripples and interictal events in vitro: what’s the difference? *Brain* 137(Pt 2), 463–485. 10.1093/brain/awt348 24390441

[B25] Kubota D. Colgin L. L. Casale M. Brucher F. A. Lynch G. (2003). Endogenous waves in hippocampal slices. *J. Neurophysiol.* 89 81–89. 10.1152/jn.00542.2002 12522161

[B26] Kupferschmidt D. A. Juczewski K. Cui G. Johnson K. A. Lovinger D. M. (2017). Parallel, but dissociable, processing in discrete corticostriatal inputs encodes skill learning. *Neuron* 96 476–489.e475. 10.1016/j.neuron.2017.09.040 29024667 PMC5663197

[B27] Kupferschmidt D. A. Lovinger D. M. (2015). Inhibition of presynaptic calcium transients in cortical inputs to the dorsolateral striatum by metabotropic GABA(B) and mGlu2/3 receptors. *J. Physiol.* 593 2295–2310. 10.1113/JP270045 25781000 PMC4457193

[B28] Li X. G. Somogyi P. Ylinen A. Buzsaki G. (1994). The hippocampal CA3 network: an in vivo intracellular labeling study. *J. Comp. Neurol.* 339 181–208. 10.1002/cne.903390204 8300905

[B29] Liang J. Xu W. Geng X. Wu J. Y. (2015). Monitoring population membrane potential signals from neocortex. *Adv. Exp. Med. Biol.* 859 171–196. 10.1007/978-3-319-17641-3_7 26238053

[B30] Lock J. T. Parker I. Smith I. F. (2015). A comparison of fluorescent Ca(2)(+) indicators for imaging local Ca(2)(+) signals in cultured cells. *Cell Calcium* 58 638–648. 10.1016/j.ceca.2015.10.003 26572560 PMC4658286

[B31] Maier N. Morris G. Johenning F. W. Schmitz D. (2009). An approach for reliably investigating hippocampal sharp wave-ripples in vitro. *PLoS One* 4:e6925 10.1371/journal.pone.0006925 PMC2732900 19738897

[B32] Maier N. Nimmrich V. Draguhn A. (2003). Cellular and network mechanisms underlying spontaneous sharp wave-ripple complexes in mouse hippocampal slices. *J. Physiol.* 550(Pt 3), 873–887. 10.1113/jphysiol.2003.044602 12807984 PMC2343079

[B33] Miyawaki T. Norimoto H. Ishikawa T. Watanabe Y. Matsuki N. Ikegaya Y. (2014). Dopamine receptor activation reorganizes neuronal ensembles during hippocampal sharp waves in vitro. *PLoS One* 9:e104438 10.1371/journal.pone.0104438 PMC4121245 25089705

[B34] Mizuseki K. Buzsaki G. (2013). Preconfigured, skewed distribution of firing rates in the hippocampus and entorhinal cortex. *Cell Rep.* 4 1010–1021. 10.1016/j.celrep.2013.07.039 23994479 PMC3804159

[B35] Muto A. Ohkura M. Abe G. Nakai J. Kawakami K. (2013). Real-time visualization of neuronal activity during perception. *Curr. Biol.* 23 307–311. 10.1016/j.cub.2012.12.040 23375894

[B36] Neher E. (2013). Quantitative aspects of calcium fluorimetry. *Cold Spring Harb. Protoc.* 2013 918–924. 10.1101/pdb.top078204 24086061

[B37] Norimoto H. Makino K. Gao M. Shikano Y. Okamoto K. Ishikawa T. (2018). Hippocampal ripples down-regulate synapses. *Science* 359 1524–1527. 10.1126/science.aao0702 29439023

[B38] Norimoto H. Mizunuma M. Ishikawa D. Matsuki N. Ikegaya Y. (2012). Muscarinic receptor activation disrupts hippocampal sharp wave-ripples. *Brain Res.* 1461 1–9. 10.1016/j.brainres.2012.04.037 22608077

[B39] Regehr W. (2000). “Monitoring presynaptic calcium dynamics with membrane-permeant indicators,” in *Imaging Neurons: A Laboratory Manual*, eds Yuste R. Lanni F. Konnerth A. (New York, NY: Cold Spring Harbor Laboratory Press).

[B40] Ross W. N. Salzberg B. M. Cohen L. B. Grinvald A. Davila H. V. Waggoner A. S. (1977). Changes in absorption, fluorescence, dichroism, and Birefringence in stained giant axons: optical measurement of membrane potential. *J. Membr. Biol.* 33 141–183. 10.1007/bf01869514 864685

[B41] Shuai J. Parker I. (2005). Optical single-channel recording by imaging calcium flux through individual ion channels: theoretical considerations and limits to resolution. *Cell Calcium* 37 283–299. 10.1016/j.ceca.2004.10.008 15755490

[B42] Sun Z. Y. Bozzelli P. L. Caccavano A. Allen M. Balmuth J. Vicini S. (2018). Disruption of perineuronal nets increases the frequency of sharp wave ripple events. *Hippocampus* 28 42–52. 10.1002/hipo.22804 28921856 PMC6047756

[B43] Svoboda K. Denk W. Kleinfeld D. Tank D. W. (1997). In vivo dendritic calcium dynamics in neocortical pyramidal neurons. *Nature* 385 161–165. 10.1038/385161a0 8990119

[B44] Wittner L. Henze D. A. Zaborszky L. Buzsaki G. (2007). Three-dimensional reconstruction of the axon arbor of a CA3 pyramidal cell recorded and filled in vivo. *Brain Struct. Funct.* 212 75–83. 10.1007/s00429-007-0148-y 17717699 PMC2662726

[B45] Xing X. Wu C. F. (2018). Unraveling synaptic GCaMP signals: differential excitability and clearance mechanisms underlying distinct Ca(2+) dynamics in tonic and phasic excitatory, and aminergic modulatory motor terminals in *Drosophila*. *eNeuro* 5:ENEURO0362-17.2018. 10.1523/ENEURO.0362-17.2018 PMC5818553 29464198

[B46] Ylinen A. Bragin A. Nadasdy Z. Jando G. Szabo I. Sik A. (1995). Sharp wave-associated high-frequency oscillation (200 Hz) in the intact hippocampus: network and intracellular mechanisms. *J. Neurosci.* 15(Pt 1), 30–46. 10.1523/jneurosci.15-01-00030.1995 7823136 PMC6578299

